# Mirabegron for intramural ureteral stones with vesical irritability: a prospective study

**DOI:** 10.3389/fphar.2025.1639032

**Published:** 2025-09-02

**Authors:** Mengjun Dai, Yuhang She, Hao Wang

**Affiliations:** ^1^ Department of Anesthesia Operating Room, The Affiliated Jiangning Hospital of Nanjing Medical University, Nanjing City, Jiangsu, China; ^2^ Department of Urology, The Affiliated Jiangning Hospital of Nanjing Medical University, Nanjing City, Jiangsu, China

**Keywords:** adrenergic beta-3 receptor agonists, mirabegron, overactive bladder, ureteral calculi, urinary tract symptoms

## Abstract

**Objective:**

To assess the efficacy of mirabegron in patients with intramural ureteral stones (6–10 mm).

**Methods:**

We prospectively randomized 92 patients with intramural ureteral stones into two groups. Patients in the mirabegron group received 50 mg of mirabegron daily, while those in the tamsulosin group received 0.4 mg tamsulosin daily. All patients were required to use the Urinary Sensation Scale (USS) to assess the urinary urgency and the Visual Analog Scale (VAS) to assess pain. Patients were followed until stone expulsion or for up to 4 weeks.

**Results:**

All of 80 patients were included in this study. 41 patients in mirabegron group and 39 patients in tamsulosin group as control. The average expulsion time was shorter in mirabegron group than in tamsulosin group (8.4 ± 2.9 vs. 11.2 ± 3.1 days, *P* < 0.0001). The stone expulsion rate (SER) was higher in mirabegron group than in tamsulosin group on 1 and 2 weeks (36.6% vs. 15.4%, *P* = 0.031 and 75.6% vs. 43.6%, *P* = 0.004). However, the SER on 4 weeks had no statistical difference between two groups (*P* > 0.05). Post-treatment VAS and USS scores were lower in mirabegron group than tamsulosin group (*P* < 0.05).

**Conclusion:**

Mirabegron not only accelerates the expulsion of intramural ureteral stones but also relieves renal colic and vesical irritability.

## Introduction

The incidence of urolithiasis has shown a continuous global increase and is one of the most common conditions encountered in urology. Ureteral stones account for approximately 20% of all urolithiasis cases, with the majority located in the distal ureter (Thongprayoon et al., 2020; Sakhaee et al., 2012; Shastri et al., 2023). If not removed in a timely manner, these stones may lead to complications such as renal colic, urinary tract infection, and progression to urosepsis (Miller and KANE, 1999; Reyner et al., 2016; Teichman, 2004; Wagenlehner et al., 2008). Patients who fail to spontaneously pass the stone often require additional interventions, depending on the stone’s characteristics and clinical presentation (Kobayashi et al., 2003; Prina et al., 2002). Both the location and size of the stone significantly influence the likelihood of spontaneous expulsion (Skolarikos et al., 2010).

The intramural ureter, due to its unique anatomical structure, represents the narrowest portion of the ureter and serves as a major barrier to spontaneous stone passage. Medical expulsive therapy (MET) has been widely used in clinical practice for facilitating stone expulsion (De Coninck et al., 2019). Stones lodged in the intramural ureter not only cause symptoms and complications typical of ureteral stones but are also frequently associated with vesical irritability—manifested as pain, urinary urgency, and frequency—similar to overactive bladder (OAB). Although tolterodine has been shown to alleviate these irritative symptoms, it does not promote stone passage (Lv and Tang, 2013; Maghsoudi et al., 2018). Therefore, an ideal therapeutic approach for intramural ureteral stones should aim to both facilitate stone expulsion and relieve vesical irritability.

Mirabegron, a β3-adrenergic receptor (β3-AR) agonist, is primarily used to treat patients with OAB (Kennelly et al., 2021; Wada et al., 2024). β3-ARs are expressed in both the urothelium and smooth muscle of the human ureter, and the inhibitory effect of β3-AR agonists on ureteral contraction has been demonstrated in several *in vivo* animal pharmacology studies (Matsumoto et al., 2013).

This study aimed to investigate whether mirabegron, as a β3-AR agonist, can effectively promote the expulsion of intramural ureteral stones while also alleviating vesical irritability.

## Methods and patients

**FIGURE 1:**
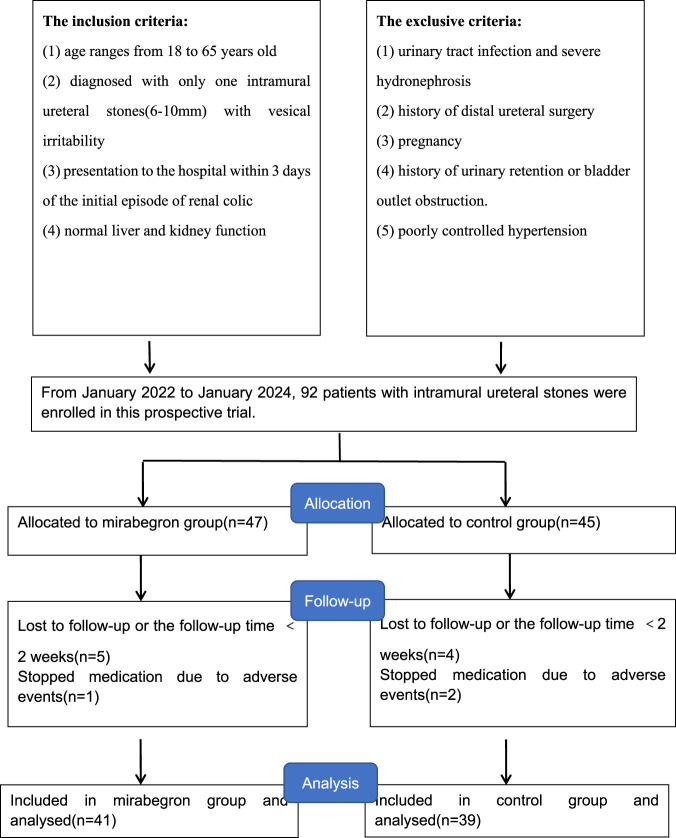
Flowchart of patient enrollment, randomization, follow-up, and analysis.

From January 2022 to January 2024, patients presenting with vesical irritability caused by intramural ureteral stones (6–10 mm in diameter) were recruited at our hospital for this study (Figure 1). The inclusion criteria included: 1) age ranges from 18 to 65 years old; 2) diagnosed with single intramural ureteral stone (6–10 mm) with vesical irritability; 3) presentation to the hospital within 3 days of the initial episode of renal colic; 4) normal liver and kidney function. Exclusion criteria included: 1) urinary tract infection and severe hydronephrosis; 2) history of distal ureteral surgery; 3) pregnancy; 4) history of urinary retention or bladder outlet obstruction; 5) poorly controlled hypertension. This study complied with the 1964 Declaration of Helsinki and received approval from the Ethics Committee of the Affiliated Jiangning Hospital of Nanjing Medical University (202100529). Routine evaluations included medical history, physical examination, blood and urine routine tests, etc.

Abdominal ultrasound, kidney-ureter-bladder (KUB) radiography, or non-contrast computed tomography (CT) were used to diagnose the intramural ureteral stones based on clinical need. Stone size was measured as the maximal diameter via KUB, ultrasonography, or CT imaging. Upon providing informed consent, patients were randomized into two groups using sealed envelopes in a 1:1 allocation ratio. The mirabegron group received 50 mg once daily, while the control group was administered 0.4 mg of tamsulosin per day. If renal colic occurred, both groups received 100 mg oral ibuprofen. Participants were adviced to intake of at least 2 L water daily and filter their urine to determine whether the stone is expelled. Medication was discontinued upon stone expulsion. Patients were followed up for 4 weeks. Patients who had not expelled their stones after 4 weeks were referred to ureteroscopic lithotripsy. Urinary urgency was using the Urinary Sensation Scale (USS) to assess. Pain intensity during stone episodes was using the Visual Analog Scale (VAS) to evaluated.

Sample size calculation ensured 95% statistical power with a 5% type I error. Continuous variables with normal distribution are showed as mean ± standard deviation (SD) and analyzed using the Student’s t-test. Categorical data are showed as counts and analyzed using the Chi-square test. Data was analyzed using SPSS v.22.0 (IBM Corp., United States). *P* < 0.05 was considered statistically significant.

## Results

92 patients were initially enrolled and randomly assigned to two groups. Nine patients lost follow-up and three patients withdrew from the study due to adverse events, all of whom recovered with symptomatic management. Finally, 80 patients included in the study: 41 in the mirabegron group and 39 in the tamsulosin group (Figure 1). Patients demographic are summarized in Table 1. No statistically differences were showed between two groups in sex, age, BMI, stone size, stone laterality, history of hypertension, smoking, or hydronephrosis grade (*P* > 0.05).

**TABLE 1:** Baseline demographics and clinical characteristics of patients.

Variable	Mirabegron (n = 41)	Control (n = 39)	*p* value
Sex, n			
Male	22	20	0.832
Female	19	19	
Age (years)	39.4 ± 5.1	40.3 ± 6.1	0.475
BMI(kg/m^2^)	24.1 ± 3.4	23.9 ± 3.6	0.799
stone size (mm)	7.9 ± 1.2	8.1 ± 1.4	0.494
Laterality, n			0.823
Left	21	19	
Right	20	20	
Hypertension history, n			0.582
No	26	27	
Yes	15	12	
Smoker, n			0.651
No	21	18	
Yes	20	21	
Hydronephrosis, n			0.100
Negative	17	20	
Mild	15	12	
Moderate	9	7	

Abbreviations: BMI, body mass index.

Note: *P < 0.05, **P < 0.01.

The clinical outcomes for both groups are shown in Table 2. After 1 and 2 weeks of pharmacological treatment, more patients achieved stone-free status in the mirabegron group than tamsulosin group (36.6% vs. 15.4%, *P* = 0.031 and 75.6% vs. 43.6%, *P* = 0.004). However, by 4 weeks, there was no statistically difference in stone expulsion rate (SER) between the two groups (92.7% vs. 89.7%, *P* = 0.709). Patients in the mirabegron group reported fewer daily episodes of pain (1.4 ± 0.6 vs. 1.8 ± 0.8, *P* = 0.013) and shorter mean stone expulsion time than those in the tamsulosin group (8.4 ± 2.9 vs. 11.2 ± 3.1, *P* < 0.0001). There were no statistical differences in USS and VAS between two groups prior to treatment (*P* > 0.05). However, both in the mirabegron group were statistical lower than those in the tamsulosin group (3.4 ± 1.2 vs. 5.7 ± 1.4, *P <* 0.0001 and 2.1 ± 0.8 vs. 3.3 ± 1.1, *P <* 0.0001). There were no statistical differences in adverse events between two groups (*P* > 0.05). After 4 weeks, patients who had not passed their stones underwent ureteroscopic lithotripsy. Intraoperative findings revealed varying degrees of ureteral edema and stricture in these patients.

**TABLE 2:** Comparison of clinical outcomes between two groups.

Outcome	Mirabegron (n = 41)	Control (n = 39)	p value
Stone expulsion Rate (SER), n (%)			
1st-week follow-up	15/41 (36.6)	6/39 (15.4)	0.031*
2nd-week follow-up	31/41 (75.6)	17/39 (43.6)	0.004**
4th-week follow-up	38/41 (92.7)	35/39 (89.7)	0.709
Renal colic episodes (per day)	1.4 ± 0.6	1.8 ± 0.8	0.013*
Stone expulsion time (days)	8.4 ± 2.9	11.2 ± 3.1	<0.0001**
Visual Analog Scale (VAS)			
Before treatment	7.2 ± 1.4	6.9 ± 1.3	0.324
After treatment	3.4 ± 1.2	5.7 ± 1.4	<0.0001**
Urinary Sensation Scale (USS)			
Before treatment	3.5 ± 1.3	3.7 ± 1.2	0.477
After treatment	2.1 ± 0.8	3.3 ± 1.1	<0.0001**
Drug unwanted effects, n			0.521
Dry mouth	1	2	
Headache	1	2	
Dizziness	1	3	
Hypertension	3	2	
Constipation	2	1	
Ejaculation dysfunction	2	0	

Abbreviations: SER, stone expulsion rate; VAS, visual analogue scale; USS, urinary sensation scale.

Note: *P < 0.05, **P < 0.01.

## Discussion

The intramural ureter, located within the muscular wall of the bladder, extends from its lateral entrance to the trigonal orifice. As the most constricted segment of the ureter, it is the primary site of stone impaction during urinary passage (Bensalah et al., 2008). Obstruction at this site often induces vesical irritability, manifesting as bladder discomfort, urinary urgency, and increased frequency of micturition (Lv and Tang, 2013).

The therapeutic principle of MET involves relaxation of ureteral smooth muscle to promote stone clearance. Pharmacological agents employed in MET include α-adrenergic receptor antagonists, calcium channel blockers, phosphodiesterase inhibitors, and antispasmodics (Türk et al., 2017). Among them, the α1-adrenergic receptor antagonist tamsulosin has demonstrated superior clinical efficacy, supported by multiple randomized clinical trials (Ye et al., 2011; Ye et al., 2018). Its mechanism involves inhibition of α1-ARs, reducing ureteral contractility and promoting luminal dilation (Kwon et al., 2015). Additionally, β2-and β3-adrenergic receptors expressed in ureteral smooth muscle and urothelium also contribute to smooth muscle relaxation.

Notably, mirabegron—a selective β3-AR agonist originally approved for the treatment of OAB—may exert off-target effects beneficial for stone expulsion (Dehvari et al., 2018). In a prospective trial, tamsulosin and mirabegron used preoperatively for semi-rigid ureterolithotripsy improved stone-free rates (SFR) and facilitated stone access, without increasing complication rates (Bayar et al., 2019). Solakhan et al. (Solakhan et al., 2019) reported that mirabegron improved the SER for intramural stones smaller than 5 mm, though not for larger stones, and noted a limited sample size. Another study by Bayar et al. (Bayar et al., 2020) suggested mirabegron had no significant effect on stone passage; however, it included only 22 cases of stones <6 mm, and did not specify their locations. Previous studies have shown that mirabegron can significantly reduce renal colic episodes (Faridi and Deshpande, 2024). Our findings are consistent with this, revealing a significant reduction in renal colic frequency among patients treated with mirabegron compared to those receiving tamsulosin. Since β3 receptors are primarily distributed in the distal ureter, persistent obstruction and smooth muscle contraction may lead to receptor downregulation, potentially compromising drug efficacy. To mitigate this, we included only patients who sought medical attention within 3 days of their initial renal colic episode, reducing the likelihood of β3-AR downregulation interfering with stone expulsion. Unlike previous studies, our investigation focused specifically on intramural ureteral stones and included a larger sample size. We found that the SER in the mirabegron group was higher than that of the tamsulosin group at 1 and 2 weeks post-treatment, although no significant difference was observed at 4 weeks. Additionally, patients in the mirabegron group had a shorter mean stone expulsion time and fewer daily renal colic episodes. These findings suggest that mirabegron, through β3-AR-mediated ureteral smooth muscle relaxation, effectively facilitates stone passage and reduces spasms. No serious complications were observed in either group, and there was no statistically significant difference in adverse events between them (P > 0.05), supporting the safety profile of mirabegron. While previous studies have explored the role of mirabegron in MET, limited research has addressed its efficacy in relieving vesical irritability associated with intramural ureteral stones. In our study, pre-treatment USS and VAS scores were comparable between groups. Post-treatment, both scores significantly reduced in both groups, with the mirabegron group showing greater reductions. This indicates that mirabegron may provide superior symptom relief for stone-related pain and irritative voiding symptoms, possibly through its relaxant effect on ureteral smooth muscle. Lu et al. (Lv and Tang, 2013) demonstrated that a combination of tamsulosin and tolterodine effectively managed intramural calculi with vesical irritability. However, combination therapy may reduce patient compliance due to pill burden. Mirabegron, as a monotherapy, may offer a more convenient alternative.

This study has several limitations. First, the follow-up duration was limited to 4 weeks, potentially overlooking long-term adverse effects. Second, the USS and VAS scores are subjective measures and may be influenced by individual perception, lacking objective validation. Third, this was a single-center study. Future multi-center studies are warranted to further evaluate the efficacy and safety of mirabegron in managing intramural ureteral stones.

## Conclusion

Mirabegron is a safe and effective option for accelerating the expulsion of intramural ureteral stones, while also alleviating pain and vesical irritability. These advantages highlight its potential for broader clinical application.

## Data Availability

The raw data supporting the conclusions of this article will be made available by the authors, without undue reservation.
